# Trail without Catheter after Transurethral Resection of Prostate: Clamp It or Not?

**DOI:** 10.1155/2016/1562153

**Published:** 2016-03-13

**Authors:** Vikash Talreja, Aun Ali, Summaya Saeed, Kiran Rani, Sunil Sadruddin Samnani, Farah Naz Farid, Mumtazuddin Haider

**Affiliations:** ^1^Department of Surgery, Jinnah Medical College Hospital, SR-6, 7/A, Korangi Industrial Area, Karachi 74900, Pakistan; ^2^Department of Obstetrics and Gynecology, Jinnah Medical College Hospital, SR-6, 7/A, Korangi Industrial Area, Karachi 74900, Pakistan; ^3^Department of Emergency, Jinnah Medical College Hospital, SR-6, 7/A, Korangi Industrial Area, Karachi 74900, Pakistan; ^4^Mind and Brain Service Line, Aga Khan University Hospital, Stadium Road, P.O. Box 3500, Karachi, Pakistan; ^5^Department of Surgery and Urology, Jinnah Medical College Hospital, SR-6, 7/A, Korangi Industrial Area, Karachi 74900, Pakistan

## Abstract

*Background.* There has been argument between clinical practitioners about clamping catheter or not prior to its removal after transurethral resection of prostate (TURP). We conducted a clinical trial to assess whether clamping has any role in early bladder tone recovery particularly in patients who undergo TURP.* Methods.* Randomized clinical trial was conducted at a tertiary care hospital, Karachi from January 2014 to July 2015. Eighty-six study participants who underwent TURP were randomly allocated into two groups of 43 participants each. In Group I, patient's Foley catheter was not clamped prior to its removal and in Group II Foley catheter was clamped. Data of all subjects were analyzed using SPSS version 20.* Results.* There was no significant difference in age and weight of resected tissues between two groups. Among 4 patients in Group I who required recatheterization, 1 patient was discharged with catheter as compared to Group II in which 2 patients were discharged with catheter (*P* = 0.99). Only 1 patient (2.3%) in Group II had bleeding which required recatheterization. Length of stay was significantly affected by early and free removal of Foley catheter (*P* < 0.001).* Conclusion.* The results of current study identified that clamping whether done or not had no significant impact on urinary retention.

## 1. Introduction

Prevalence of Benign Prostatic Enlargement (BPE) increases in advanced age. Most of these patients present with lower urinary tract infections [1]. Available literature suggests that average length of stay for patients undergoing Transurethral Resection of Prostate (TURP) is 2–7 days and average cost of accommodation accounts for 29–33%. So any method which could minimize length of hospital stay will significantly reduce cost of healthcare resources [2]. Increased length of hospital stay increases the cost of TURP as compared to newer modification in surgeries for patients having BPE [3].

So far there has been no agreement between clinical practitioners that clamping the catheter draining tubes prior to its removal influences patients' outcomes compared to free-draining of catheters [4]. Previous studies have also shown that clamping of catheter draining tubes had no effect on postoperative bladder dysfunction [5]. We certainly have limited evidence to say if any significance lies in clamping of urinary catheters. For this purpose we conducted a clinical trial to assess whether clamping has any role in early bladder tone recovery particularly in patients who underwent TURP.

## 2. Material and Methods

A randomized clinical trial was conducted at a tertiary care hospital, Karachi, from January 2014 to July 2015. Eighty-six study participants who underwent TURP were randomly allocated into two groups. Group I included those patients whose Foley catheter was not clamped prior to its removal and Group II included those whose Foley catheter was clamped. Clamping refers to interrupting bladder flow by obstructing the drainage pipe of Foley catheter and releasing it intermittently as patient feels urge to void.

All patients who got admitted for TURP during the period were recruited in the study. After performing thorough history and examination, all participants underwent investigations including urine analysis and culture, baseline biochemistry investigations, ultrasound imaging, and uroflowmetry. Written and informed consent was taken, and confidentiality of the patients was taken into account. A total of 3 participants had positive urine culture for which they were given antibiotics according to culture and sensitivity report, whereas remaining patients were given one dose of third-generation cephalosporin in preoperative period.

Patients who presented with history of trauma to spinal cord and cerebrovascular accidents and patients having comorbid conditions like diabetes mellitus or any other urogenital problems such as urethral strictures were excluded from the study.

A structured questionnaire was designed and filled in by the admitting doctor. Foley catheter was removed once patient got mobilized, passed stool, and had no active bleeding or infection. Foley catheter was removed in the early morning in all cases.

Questionnaires were collected; data of all subjects who were included in the study was entered and analyzed using SPSS version 20. A *P* value of ≤0.05 was considered as significant. And a confidence interval of 95% was taken into account.

## 3. Results

The results of randomized clinical trial were based on effect of removing free drainage Foley catheter in comparison to clamping of Foley catheter prior to its removal. In total, 86 patients were included in the study. Participants were randomized into groups on the basis of removal of Foley catheter without clamping (Group I) and removal of Foley catheter after clamping (Group II). All participants were randomized into two equal groups of 43 participants each. As shown in Table 1, mean age of study participants was 64.21 years (±5.36 SD) in Group I and 63.05 years (±4.69 SD) in Group II. There was no significant difference in age of study participants between two groups (*P* = 0.28). In like manner, difference in weight of resected tissue was also found to be statistically insignificant in two groups (*P* = 0.87). Around 5 patients (11.6%) in Group I went into urinary retention, whereas 3 patients (7%) in Group II underwent retention. In Group I, 4 out of 5 patients underwent recatheterization as compared to Group II where 2 of 3 patients required recatheterization (*P* = 0.68). Only 1 patient from each group was found to have urinary tract infection despite of being on antibiotic (*P* = 1.00). Patient with UTI was treated with antibiotic based on repeated culture and sensitivity test of urine. Among 4 patients in Group I who required recatheterization, 1 was discharged with catheter as compared to Group II in which both patients who were recatheterized were discharged with catheter (*P* = 0.99). However, from 2 patients of Group II, 1 patient (2.3%) was recatheterized due to bleeding. Length of stay was significantly affected by early and free removal of Foley catheter as patient in Group I had average length of stay of 2.6 days (±0.82 SD) and Group II patients had 4.56 days (±1.36 SD).

## 4. Discussion

TURP is a conventionally used method for patients with BPE. A Foley catheter is used in all patients who undergo TURP. However, there is still insufficient literature available regarding the appropriate time for catheter removal [2]. Catheterization in patients following TURP helps to prevent bleeding, monitor urine output, provide comfort in urination, and reduce the symptoms of urethral irritation. Several studies suggested and recommended early removal of Foley catheter in post-TURP patients [6]. Nakagawa and Toguri and other studies reported that early removal of Foley catheter not only reduces the hospital stay but also proved to be cost effective for the patients. Besides these benefits, catheterization for a short period of time also minimizes the chances of urinary tract infections [2, 6–8]. The measures that assist in reducing length of stay eventually impact financial outcome. Since 1987 to 1995 mean stay of patients had been greatly reduced from 10.6 to 6.1 days [9]. Another study reported that patients developed no significant complications within the first 24 hours after Foley catheter removal [10]. In line with the finding of the current study, Oberst et al. during their study observed that removal of catheter following free drainage or clamping was not associated with postoperative voiding dysfunction [11]. Moreover, in our study patients who underwent early removal of Foley catheter without clamping had shorter duration of stay, thus decreasing cost of healthcare resources. Similar to the evidences established in the literature, in the current study the resected prostate gland size was not associated with the duration of catheterization [3].

In current study, among patients whose Foley catheter was removed without clamping 4 patients were recatheterized because of severe urge for urination and palpable urinary bladder. Around 90% of the participants were discharged without Foley catheter as compared to another study, where 80% of patients were discharged without Foley catheter with reduced duration of stay [12]. In line with previous study no significant difference was observed for recatheterization in patients undergoing clamping versus free drainage of Foley catheter.

## 5. Conclusion

The current study highlights that clamping whether done or not had no significant impact on urinary retention. Still in many healthcare settings in Pakistan clamping of Foley catheter is being practiced. Removal of Foley catheter without clamping had significantly decreased length of stay and thus reduced cost of healthcare resources.

## Figures and Tables

**Table 1:** Comparison between Group I and Group II.

	Foley removal without clamping (*n* = 43)	Foley removal with clamping (*n* = 43)	*P* value
Catheter removal			
Successful	38 (88.4)	40 (93)	
Unsuccessful	5 (11.6)	3 (7)	
Age (years)	64.21 ± 5.36	63.05 ± 4.69	0.28
Weight of resected tissue (grams)	19.14 ± 6.50	19.42 ± 9.65	0.87
Urinary retention			
Developed	5 (11.6)	3 (7)	0.71
Not developed	38 (88.4)	40 (93)	
Urinary retention; recatheterization	4 (9.3)	2 (4.7)	0.68
Discharge with catheter	1 (2.3)	2 (4.7)	0.99
Urinary tract infection requiring recatheterization	1 (2.3)	1 (2.3)	1.00
Bleeding requiring recatheterization	0 (0)	1 (2.3)	0.99
Length of stay (days)	2.6 ± 0.82	4.56 ± 1.36	<0.001
